# Long-term outcomes among localized prostate cancer survivors: prospective predictors for return-to-work three years after cancer rehabilitation

**DOI:** 10.1007/s00520-021-06376-6

**Published:** 2021-08-15

**Authors:** Anneke Ullrich, Hilke Maria Rath, Ullrich Otto, Christa Kerschgens, Martin Raida, Christa Hagen-Aukamp, Corinna Bergelt

**Affiliations:** 1 Department of Medical Psychology, University Medical Center Hamburg-Eppendorf, Martinistr. 52, 20246 Hamburg, Germany; 2 Rehabilitation Clinics Hartenstein GmbH, Clinic Quellental, Bad Wildungen, Germany; 3 Vivantes Rehabilitation Clinic GmbH, Berlin, Germany; 4 HELIOS Rehabilitation Clinic Bergisch-Land, Wuppertal, Germany; 5 Niederrhein Rehabilitation Clinic, Korschenbroich, Germany

**Keywords:** Prostate cancer, Employment, Long term, Rehabilitation, Return to work, Survivorship

## Abstract

**Purpose:**

This study aimed at (**1**) investigating the work status of men treated by radical prostatectomy due to diagnosis of localized prostate cancer (LPCa) three years after having attended a cancer rehabilitation program and (**2**) identifying prospective risk factors for not working at this time point.

**Methods:**

In a longitudinal, questionnaire-based multicenter study, 519 working-age LPCa survivors reported on their work status 12 and 36 months following rehabilitation. Chi-square tests/*t* tests and multivariable logistic regression analysis were used to identify prospective factors associated with not working at 36 months follow-up.

**Results:**

Nearly three quarter of LPCa survivors (*N* = 377, 73%) worked 3 years after post-acute rehabilitation. Most participants (*N* = 365, 71%) showed continuous return-to-work (RTW) patterns as they worked both 1 and 3 years following rehabilitation. Multivariable regression analysis revealed older age, low or middle socio-economic status as well as resigned and unambitious work behavior and fatigue at the time of attending the rehabilitation program to be prospective factors for not working at 36 months follow-up. Low socio-economic status [Odds ratio (OR) 4.81, 95% confidence interval (CI) 2.07–11.16] and unambitious work behavior [OR 4.48, 95% CI 2.16–9.31] were the strongest predictors.

**Conclusion:**

Long-term work retention is a realistic goal among LPCa survivors. The results contribute to the identification of at-risk LPCa survivors early in the RTW process. Special attention should be paid to social inequality. Further, interventions related to the management of fatigue and work-related coping styles could improve long-term RTW, as these were relevant, but potentially modifiable factors impeding work retention.

## Introduction

Early detection and improved cancer treatments have led to an increasing number of cancer survivors. For those in working age, return-to-work (RTW) has become a key issue for national cancer control plans across Europe [26]. Research has demonstrated manifold individual and societal values of work reintegration in cancer survivors [44], and many cancer survivors are able to continue working or return to work [28]. Nevertheless, adverse effects of the disease and treatment may have long-term effects, including drop-out from the labor market after initial RTW or loss of work productivity [2].

There is an increasing body of research focusing on RTW outcomes of cancer survivors 2 years or more after diagnosis. A recent meta-analysis based on 21 studies from 2000–2019 reported a pooled estimate of prevalence for work retention of 0.73 for 14,207 cancer survivors 2–14 years post-diagnosis [12]. Most studies focused on women with breast cancer compared with one study focusing on men with prostate cancer; however, cancer site had no impact on the pooled estimate [12]. Two to six years after diagnosis, 676 younger cancer survivors from the USA (aged 28–54 years, 213 males, multiple cancer sites) showed lower employment rates and weekly working hours compared with similarly aged non-cancer controls [30]. Among 25,094 Danish cancer survivors (11,764 males, multiple cancer sites) who had remained employed 4 years after diagnosis, cancer had a stronger negative impact on the work status when pre-cancer skills requirements included high levels of manual skills or low levels of cognitive skills [19]. Beyond RTW rates, studies demonstrate detrimental cancer-related work changes in long-term survivors [3, 29]. For example, a German population-based study with 1558 cancer survivors (377 males, multiple cancer sites) showed that 17% of returnees (9% in the subgroup of prostate cancer) had reduced their working hours within 5 years after work resumption [3].

To restore work ability and support sustained reintegration into working life after cancer, rehabilitation is a critical component of cancer care with multidisciplinary approaches being most effective [11, 35]. Based on social legislation, cancer patients in Germany are entitled to attend rehabilitation programs if explicit criteria are met. Such 3-week programs follow a multimodal therapeutic approach and are mostly provided in inpatient rehabilitation clinics. Costs are mainly covered by pension and health insurance [20].

As cancer sites are associated with different physical, psychological and social sequelae, research on RTW outcomes should consider specific cancer survivor groups. As for prostate cancer, a systematic review has shown treatment side effects such as urinary incontinence and fatigue to be associated with reduced work status and reduced work ability; hence, these are specific factors that could prevent men with prostate cancer from returning to work [27]. In a scoping review on prostate cancer treatment and work, urinary continence was identified as a major factor associated with work resumption [47]. However, for survivors of (localized) prostate cancer, longitudinal studies on work outcomes exceeding a follow-up period of 2 years remain scarce [27, 47]. Therefore, the aims of our study were (1) to investigate work retention of cancer survivors treated by radical prostatectomy (RP) due to diagnosis of localized prostate cancer (LPCa) 3 years after having attended a post-acute cancer rehabilitation program and (2) to identify prospective risk factors for not working at this point in time.

## Methods

### Setting and participants

This multicenter, prospective longitudinal study was designed to evaluate RTW outcomes and psychosocial well-being of LPCa survivors who attended a rehabilitation program immediately following RP. The study comprised four points of measurement: at the beginning (t1, baseline) and at the end of the rehabilitation program (t2) and at 12 months (t3) and 36 months (t4) follow-ups. In previous publications on this study, we have reported about work-related problems these cancer survivors faced up to 1 year post-rehabilitation [40, 41]. The present manuscript focuses on RTW outcomes 3 years after having attended the cancer rehabilitation program.

Participants had been consecutively recruited in 4 German specialized rehabilitation clinics during the initial clinical consultation at the beginning of the rehabilitation program between October 2010 and June 2012. Inclusion criteria were LPCa (no evidence of lymphogenic and distant metastasis), start of the rehabilitation program within 14 days after the end of acute treatment (“post-acute rehabilitation”), working age (18–64 years) at study enrolment, and paid work prior to RP. Exclusion criteria were early retirement or pending application for a pension, severe psychological/physical stress (physician’s assessment), and language problems.

The first two questionnaires (t1, t2) were handed over by the treating physicians including information on data collection at follow-up and its relevancy. Follow-up questionnaires (t3, t4) were sent by mail including a single reminder after 4 weeks. Medical data were provided by physicians and retrieved from medical records during the rehabilitation program.

The study protocol was reviewed and approved by the ethics committee of the General Medical Council of Hamburg (PV3547), and the department of data security of the German Pension Insurance Agency in Berlin, Germany.

### The rehabilitation program

All LPCa survivors received a post-acute (non-study-specific) multidisciplinary medical rehabilitation program with high treatment intensity that is based on guidelines concerning cancer rehabilitation [9]. Clinics offered either inpatient and/or full-time outpatient cancer rehabilitation. Both in- and outpatient rehabilitation programs include psychological support/therapy, patient education, medical treatment, physical training, and social counseling. Categories of therapeutic treatment are listed in the Pension Insurance’s KTL classification system [13]. Inpatients and outpatients received a comparable treatment dose of approximately 12 hours per week, but to some extent, the type of treatments differed [32]. Largest group differences were found in the category “ergotherapy, occupational therapy and other functional therapies” in favor of inpatients and in the category “sports and exercise therapy” in favor of outpatients [32]. Discrepancies were mainly due to differences regarding patients’ characteristics in the rehabilitation settings [32].

### Measures

#### Variables on RTW outcomes

Work status was assessed by LPCa survivors’ self-report at 12 months follow-up (t3) and 36 months follow-up (t4). Participants confirmed one of the following answering options: paid part- or full-time employment, unemployment, disability pension, or retirement. Retired participants additionally reported the date of retirement at both measurement points to differentiate between early retirement, defined as having been retired at younger age than regular retirement age, and regular old-age retirement.

Beyond their work status, participants were asked whether they had experienced changes of their work situation due to their cancer diagnosis within the last year including job tasks and/or the workplace, weekly working hours, and interpersonal relationships at work.

#### Covariates

The set of potential predictors was multidimensional and reflected the seven dimensions of factors influencing RTW after cancer as proposed in the model of Feuerstein et al. [18]. Variables were measured during the rehabilitation program (t1 or t2) reflecting a recovery stage oriented to return to work.

- Factors related to *personal characteristics/socio-demographics:* At t1, LPCa survivors reported on demographics. The socio-economic status was categorized into low, middle, and high using an indicator-based approach applied in the German National Health Survey (“Winkler Index”) [45].
- Factors related to *health status and well-being:* At t1, physicians provided information on the date of first diagnosis via punch biopsy, tumor stage, RP procedure, and comorbidities. The extent of urinary incontinence (study-specific measure) and the Karnofsky Performance Status [23] was assessed at t2.
- Factors related to the *healthcare system:* The rehabilitation setting (inpatient vs. outpatient) was retrieved from medical records at t1.
- Factors related to *functioning* and *symptoms:* At t2, health-related quality of life was measured by the European Organization for Research and Treatment of Cancer Quality of Life Questionnaire EORTC QLQ-C30 [1] and the prostate-specific module EORTC QLQ-PR25 [42]. Higher scores (scale range 0–100) reflect either higher levels of functioning or higher symptom burden. Both questionnaires show good psychometric properties [1, 42]. Anxiety and depression were assessed by the Hospital Anxiety and Depression Scale (HADS) [22] with its two subscales, each ranging from 0 to 21 with values ≥ 11 indicating clinically relevant symptom levels. Studies show high validity and reliability of the HADS [21].
- Factors related to *work demands* and *work environment:* Information on work-related issues were collected at t1. Potential risk factors for early retirement were assessed by the Screening Instrument Work and Occupation (German abbreviation: SIBAR) [8]. It has been specifically designed for the rehabilitation context and authors report good reliability and validity [8]. Reciprocity of work-related effort and reward was measured by the Effort-Reward Imbalance at Work Questionnaire (ERI) [34], which shows good psychometric properties and clinical usefulness [34]. Imbalance is diagnosed if the ratio of effort and reward equals or exceeds 1. Work-related behavior patterns and coping styles were assessed by the Occupational Stress and Coping Inventory (German abbreviation: AVEM) [33], which classifies work behavior patterns based on the ways a person handles stressful situations. It differentiates four types: the healthy-ambitious type (G: “Good health”), the unambitious type (S: “Attitude of sparing investment at work”), the excessively ambitious type (Risk pattern A: “Ambitious”), and the resigned type (Risk pattern B: “Burnout”). The AVEM has been reported to be valid and reliable in non-clinical and clinical populations including cancer patients [31, 33].

### Recruitment procedures and nonresponder analysis

#### Recruitment

**Fig. 1:**
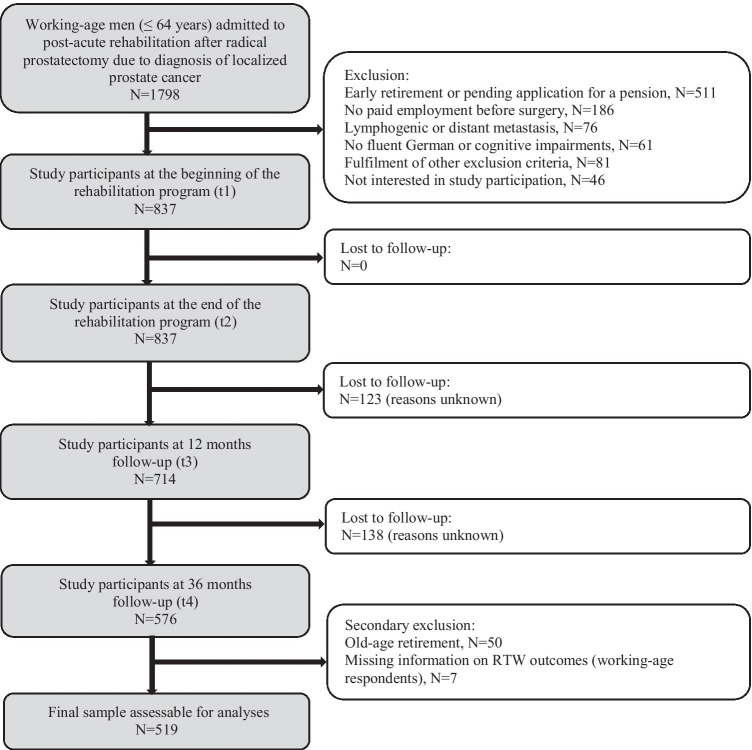
Study recruiting process and sample development

The patient flow diagram is displayed in Fig. 1. Of 1798 patients admitted to the rehabilitation clinics, 883 (49.1%) eligible patients were approached for study participation, of which 837 (94.7%) consented to participate. Among those, 837 (100%) answered questionnaires at t1 and t2, 714 (85%) at 12 months follow-up (t3), and 576 (81% of 714) at 36 months follow-up (t4). At t4, 50 participants had reached the regular retirement age during follow-up (65–67 years, depending on the year of birth), and had to be excluded from the analyses on RTW outcomes. Further, seven working-age participants did not report RTW outcomes, leaving 519 LPCa survivors for analyses.

#### Nonresponder analysis at 36 months follow-up

Marriage (85 vs. 76%), higher income (≥ 3000 €: 49 vs. 39%), and endoscopic rather than robot-assisted RP (51 vs. 47%) at t1 were significantly more frequent in respondents than nonrespondents at 36 months follow-up. Further, depression (HADS-D ≥ 11: 6 vs. 12%) and anxiety (HADS-A ≥ 11: 12 vs. 17%) at t1 were significantly less frequent in respondents (*p* = 0.001–0.014).

### Statistical analysis

According to their work status, working-age LPCa survivors (*N* = 519) were stratified into two groups at each follow-up: “working” (working part- or full-time) or “not working” (covering the remaining categories). Depending on the metrics of the variables, chi-square tests (or Fisher’s exact test) and two-sample *t* tests were conducted to compare the two groups with regard to factors related to personal characteristics, health status and healthcare system, functioning and symptoms, as well as work-related issues. We constructed a multivariable binary logistic regression model including all factors that significantly differentiated between the two groups in bivariable analyses. Regression analysis was conducted with “not working at 36 months follow-up” being the dependent variable (reference group: working). Based on the theoretical and statistical pre-selection, potential predictors were entered simultaneously into the multivariable regression model (method: enter). Multicollinearity analysis showed variance inflation factors of less than 1.4; hence it did not pose a problem [25]. Missing data was handled by list-wise deletion, resulting in a final sample of 505 out of 519 (97%) LPCa survivors. Strengths of associations were expressed as odds ratios (OR) with 95% confidence intervals (CI). To evaluate the goodness-of-fit of the logistic model we used Nagelkerke’s pseudo R^2^ with values > 0.5 being considered very good [5].

Analyses were performed using SPSS software version 22.0 (IBM, 2013). All significance tests were two-tailed using a significance level of α < 0.05.

The Strengthening the Reporting of Observational Studies in Epidemiology (STROBE) [43] guidelines were used to ensure the reporting of this observational study.

## Results

### Sample characteristics

Of 519 LPCa survivors, 85% were married, 46% low-educated, and baseline age was on average 57 years. The majority had been diagnosed with stage T1-T2 prostate cancer within 3 months prior rehabilitation (Table 1).

**Table 1:** Factors related to personal characteristics, health status, and healthcare system of the entire sample and with regard to work status at 36 months follow-up (*N* = 519)

Factors related to the dimensions of *“Personal characteristics / socio-demographics”*, *“Health status and well-being”*, and *“Healthcare system”*	Entire sample (*N* = 519)		Working (*N* = 377)		Not working (*N* = 142)				
	n	%	n	%	n	%	T/χ^2^	df	p
Factors related to the dimension of *“Personal characteristics / socio-demographics”*									
Age (M, SD) (t1)	519	56.5 (4.0)	377	55.3 (4.9)	142	59.6 (1.9)	-12.474	484.351	**< .001**^a^
Family status (t1)									
Single	32	6.2	26	6.9	6	4.3	1.232	2	.540^b^
Married	437	85.0	317	84.5	120	86.3			
Separated, divorced, widowed	45	8.8	32	8.5	13	9.4			
School education (t1)									
Up to 9 years	234	46.3	159	42.9	75	56.0	10.689	2	**.008**^b^
10 years	115	22.8	84	22.6	31	23.1			
12–13 years	156	30.9	128	34.5	28	20.9			
Occupational position (t1)									
Blue-collar job	188	36.5	129	34.6	57	40.7	10.947	2	.228^b^
White-collar job	257	49.9	188	50.4	69	49.3			
Self-employed or public servant	70	13.6	56	15.0	14	10.0			
Monthly household net income (t1)									
< 2000 €	79	16.0	51	14.0	28	21.2	8.540	3	**.036**^b^
2000- < 3000 €	180	36.3	128	35.3	52	39.4			
3000- < 4000 €	140	28.3	104	28.7	36	27.3			
4000 € or more	96	19.4	80	22.0	16	12.1			
Socio-economic status (t1)									
Low	97	18.9	62	16.6	35	25.2	11.688	2	**.003**^b^
Middle	284	55.4	202	54.0	82	59.0			
High	132	25.7	110	29.4	22	15.8			
Factors related to the dimensions of *“Health status and well-being”* and *“Healthcare system”*									
Tumor stage at diagnosis (t1)									
T1/T2	423	81.5	315	83.6	108	76.1	3.847	1	.050^b^
T3	96	18.5	62	16.4	34	23.9			
Time since diagnosis by punch biopsy (t1)									
0–3 months	456	87.9	329	87.3	127	89.4	.455	1	.500^b^
≥ 4 months	63	12.1	48	12.7	15	10.6			
Surgical procedure (t1)									
Retropubic	267	51.4	188	49.9	79	55.6	1.723	-	.647^c^
Perineal	5	1.0	4	1.1	1	0.7			
Endoscopic	84	16.2	61	16.2	23	16.2			
Robot-assisted (DaVinci)	163	31.4	124	32.9	39	27.5			
Karnofsky performance status^d^ (t1)									
≤ 70%	184	35.5	137	36.4	47	33.1	.557	3	.906^b^
80%	234	45.1	168	44.6	66	46.5			
90%	75	14.5	54	14.3	21	14.8			
100%	26	5.0	18	4.8	8	5.6			
Comorbidities (t1)									
None	203	39.1	149	39.5	54	38.0	2.530	2	.282^b^
1	187	36.0	141	37.4	46	32.4			
≥ 2	129	24.9	87	23.1	42	29.6			
Urinary incontinence^e^ (t2)									
°0	255	49.3	192	51.2	63	44.4	4.167	3	.244^b^
°I	172	33.3	115	30.7	57	40.1			
°II	49	9.5	37	9.9	12	8.5			
°III	41	7.9	31	8.3	10	7.0			
Rehabilitation setting (t1)									
Inpatient	462	89.0	336	89.1	126	88.7	.016	1	.899^b^
Outpatient	57	11.0	41	10.9	16	11.3			

^a^Two-sample *t* test (two-tailed)

^b^Chi-square test

^c^Fisher’s exact test

^d^100%: normal activity, no complaints; 90%: able to carry on normal activities, minor signs or symptoms of disease; 80%: normal activity with effort; 70%: care for self, but unable to carry on normal activity or to do active work

^e^Grade 0: no incontinence; Grade 1: only at afternoon; Grade 2: already before noon; Grade 3: also at nights

Abbreviations: M, mean; SD, standard deviation; p, probability of type I error; T, T-statistics of the two-sample *t *test; **χ**^**2**^, Chi^2^-statistics of the chi-square test; df, degrees of freedom; t1, beginning of the rehabilitation program; t2, end of the rehabilitation program

Significant values are marked in bold

### RTW rate at 36 months follow-up and work sustainability

Three-hundred seventy-seven LPCa survivors (72.6%) worked at 36 months follow-up. Reasons for not working were early retirement in 113 (21.8%), being unemployed in 23 (4.4%) and receiving disability pension in 6 (1.2%) cases. Allocation to the “early retirement” group was defined by being retired but younger than the regular retirement age adjusted to the year of birth, and not indicating that retirement was cancer-related. Mean age of the “early retirement” group of 113 LPCa survivors was 62.7 years (SD 1.7) at follow-up.

Patterns of occupational activity were assessed by comparison of working-age LPCa survivors’ work status at 12 months (t3) and 36 months (t4) follow-up. Three-hundred sixty-five (70.7%) worked at both points of time, indicating a majority of LPCa survivors with sustained RTW. In contrast, 44 (8.5%) reported continuous occupational inactivity. In 95 (18.4%) LPCa survivors, a negative change of their work status was observed, as they worked at 12 months but no longer at 36 months follow-up. Reversely, a minority of 12 (2.3%) showed a positive change, as they did not work at first but at second follow-up.

Among 377 LPCa survivors who worked at 36 months follow-up, most reported no cancer-related changes in their work situation within the last year: 322 (88.3%) reported a stable workplace and/or job tasks, and 335 (90.8%) reported no changes of hours worked. Additionally, changes of interpersonal relationships at work were only observed in smaller subgroups of 27 (7.3%) LPCa survivors regarding relationships with employers, 21 (5.7%) with supervisors, and 20 (5.4%) with co-workers.

### Factors associated with work status at 36 months follow-up

Bivariable analyses revealed that single aspects of the applied model of work and cancer[18] significantly differed between LPCa survivors who were “working” vs. “not working” at 36 months follow-up. With regard to *personal characteristics/socio-demographics*, those of the “not working” group were significantly older (mean 59.6 vs. 55.3 years) and showed lower levels of education, monthly household net income, and socio-economic status at the beginning of the rehabilitation program (t1). Family status and occupational position were not associated with work status at 36 months follow-up (Table 1).

In contrast, no significant associations between work status and factors reflecting the *health status and well-being* and *healthcare system* were observed, including the type of RP surgery and in-/outpatient rehabilitation setting. However, LPCa survivors with tumor stage T3 showed a higher tendency of not working (Table 1).

Regarding factors related to *functioning* and *symptoms*, LPCa survivors of the “not working” group showed significantly lower physical functioning and higher levels of fatigue as assessed at the end of the rehabilitation program (t2). However, mean differences in both scales were less than 10 points, with ≥ 10 points being widely regarded as clinically important for the EORTC QLQ-C30. For most cancer-related functioning and symptom scales (EORTC QLQ-C30) and all prostate-specific symptoms (EORTC QLQ-PR25) as well as anxiety/depression (HADS), no significant differences were observed (Table 2).

**Table 2:** Factors related to functioning and symptoms of the entire sample and with regard to work status at 36 months follow-up (*N* = 519)

Factors related to the dimensions of *“Functioning”* and *“Symptoms”*	Entire sample (*N* = 519)		Working (*N* = 377)		Not working (*N* = 142)				
	M	SD	M	SD	M	SD	T	df	P^a^
Anxiety and depression (HADS, t2)									
Anxiety	4.8	3.5	4.6	3.5	5.2	3.6	- 1.599	517	.110
Depression	4.0	3.2	3.9	3.0	4.1	3.5	- .701	517	.483
Cancer-specific health-related quality of life (EORTC QLQ-C30, t2)									
Global health status	65.6	17.2	66.0	16.7	64.6	18.6	.829	517	.407
Physical functioning	75.5	16.7	68.6	21.1	69.5	18.0	2.403	517	**.014**
Role functioning	53.6	28.4	53.7	28.5	53.4	28.1	.567	517	.912
Emotional functioning	76.8	20.4	77.6	20.2	74.7	20.6	1.448	517	.148
Cognitive functioning	83.8	19.3	84.4	18.8	82.0	20.4	1.240	517	.215
Social functioning	67.9	23.6	68.2	23.3	66.9	24.3	.566	517	.572
Fatigue	33.2	21.5	31.8	21.1	37.1	22.1	- 2.528	517	**.012**
Vomiting	1.7	7.4	1.4	5.2	2.5	11.4	- 1.102	163.520	.272
Pain	24.0	25.4	23.3	25.5	25.4	25.2	- .803	517	.423
Dyspnea	16.5	22.7	15.9	23.0	18.1	22.0	- .987	516	.324
Insomnia	29.3	30.7	27.7	29.7	33.6	32.9	- 1.930	516	.054
Appetite loss	5.7	15.3	5.3	14.8	6.8	16.6	- .995	517	.320
Constipation	9.3	20.2	8.9	19.4	10.3	22.2	- .702	517	.483
Diarrhea	4.4	14.0	4.9	14.5	3.3	12.7	1.218	286.721	.224
Financial problems	24.2	29.4	25.0	29.6	22.1	28.9	1.012	516	.312
Prostate cancer-specific health-related quality of life (EORTC QLQ-PR25, t2)^b^									
Urinary incontinence	33.3	17.4	32.8	17.3	34.7	17.8	- 1.123	515	.262
Bowel symptoms	5.4	8.9	5.4	9.0	5.4	8.3	.036	509	.971
Hormonal-therapy induced symptoms	12.9	11.3	12.6	10.8	13.5	12.7	- .709	217.374	.479
Bother due to incontinence aids^c^	36.9	31.9	37.8	31.8	34.5	31.1	.814	317	.416

^a^Two-sample *t* test (two-tailed)

^b^Functioning scales omitted

^c^Entire sample: N = 319; Working at 36 months follow-up: *N* = 233; Not working at 36 months follow-up: *N* = 142; lower N because only a subgroup of patients used incontinence aids

Abbreviations: M, mean; SD, standard deviation; p, probability of type I error; T, T-statistics of the two-sample *t *test; df, degrees of freedom; t2, end of the rehabilitation program; HADS, Hospital Anxiety and Depression Scale; EORTC QLQ-C30, European Organization for Research and Treatment of Cancer Quality of Life – core questionnaire; EORTC QLQ-PR25, European Organization for Research and Treatment of Cancer Quality of Life Questionnaire – prostate-specific module

Significant values are marked in bold

With respect to factors reflecting w*ork demands* and *work environment*, LPCa survivors of the “not working” group exhibited significantly less healthy-ambitious (Type G) but more unambitious (Type S) work behavior patterns than their counterparts at the beginning of the rehabilitation program (t1). Further, those who had reported their intention to apply for a disability pension at this early time in the RTW process more frequently belonged to the “not working” group at 36 months follow-up (t4). However, LPCa survivors of this group had not reported worse self-assessed work ability, more occupational stress, or effort-reward-imbalance at t1 (Table 3).

**Table 3:** Factors related to work demands and work environment of the entire sample and with regard to work status at 36 months follow-up (*N* = 519)

Factors related to the dimensions of *“Work demands”* and *“Work environment”*	Entire sample (*N* = 519)		Working (*N* = 377)		Not working (*N* = 142)				
	n	%	n	%	n	%	χ^2^	df	P^a^
Work behavior patterns and coping styles (AVEM, t1)									
Healthy-ambitious (Type G)	133	25.6	107	28.4	26	18.3	15.313	4	**.004**
Unambitious (Type S)	170	32.8	109	28.9	61	43.0			
Excessively ambitious (Risk Tye A)	91	17.5	71	18.8	20	14.1			
Resigned (Risk Type B)	84	16.2	56	14.9	28	19.7			
Unclear	41	7.9	34	9.0	7	4.9			
Risk factors for early retirement (SIBAR, t1)									
Self-assessed work ability									
No work ability	124	24.0	84	22.4	40	28.2	1.887	2	.389
Limited work ability	342	66.2	253	67.5	89	62.7			
Full work ability	51	9.9	38	10.1	13	9.2			
Intention to apply for a disability pension (yes)	122	23.5	71	19.1	51	36.7	17.255	1	**< .001**
Occupational stress (yes)	70	13.6	46	12.3	24	17.0	1.976	1	.160
Effort-reward imbalance (ERI Cut off ≥ 1, t1)	52	10.2	40	10.7	12	8.8	.411	1	.521

^a^Chi-square test

Abbreviations: M, mean; SD, standard deviation; p, probability of type I error; **χ**^2^, Chi^2^-statistics of the chi-square test; df, degrees of freedom; t1, beginning of the rehabilitation program; AVEM, Occupational Stress and Coping Inventory; ERI, Effort-Reward Imbalance at Work Questionnaire; SIBAR, Screening Instrument Work and Occupation

Significant values are marked in bold

### Prospective predictors of not working at 36 months follow-up

Multivariable logistic regression revealed six predictors increasing the probability of not working 3 years after having attended a cancer rehabilitation program: Older age [OR  1.95; 95% Confidence Interval (CI) 1.69–2.25], low- [OR  4.81; 95% CI 2.07–11.16], or middle socio-economic status [OR  3.44; 95% CI 1.74–6.83], higher symptom burden due to fatigue reported at the end of the rehabilitation program [OR  1.02; 95% CI 1.02–1.03] as well as having expressed “unambitious (Type S)” [OR  4.49; 95% CI 2.16–9.32] or “resigned (Type B)” [OR  2.77; 95% CI 1.17–6.54] work behavior patterns at the beginning of the rehabilitation program. With few predictors, the model explained 55% of the total variability of long-term RTW (Nagelkerke’s pseudo R^2^: 0.545) (Table 4).

**Table 4:** Results of the multivariable regression model for not working at 36 months follow-up

	Not working 36 months after the end of the rehabilitation program				
	β	SE	p	OR	95% CI
Factors related to *“Personal characteristics / Socio-demographics”* (t1)					
Age	.666	.073	** < .001**	1.947	1.687–2.246
Socio-economic status					
High	Ref				
Middle	1.236	.350	** < .001**	3.442	1.735–6.829
Low	1.571	.429	** < .001**	4.811	2.074–11.158
Factors related to *“Health status and well-being”* (t1)					
Tumor stage^a^					
T3	Ref				
T1/T2	-.612	.342	.074	.542	.278–1.060
Factors related to *“Functioning”* and *“Symptoms”* (t2)					
Fatigue (EORTC QLQ-C30)	.018	.008	**.028**	1.018	1.002–1.034
Physical functioning (EORTC QLQ-C30)	-.010	.010	.306	.990	.970–1.010
Factors related to *“Work demands”* and *“Work environment”* (t1)					
Work-related behavior patterns and coping styles (AVEM)					
Healthy-ambitious (Type G)	Ref				
Unambitious (Type S)	1.501	.373	**< .001**	4.485	2.160–9.315
Excessively ambitious (Risk Type A)	.315	.430	.464	1.370	.590–3.181
Resigned (Risk Type B)	1.018	.439	**.020**	2.766	1.170–6.538
Unclear	.316	.592	.594	1.371	.430–4.375
Intention to apply for a disability pension (SIBAR)					
No	Ref				
Yes	.480	.297	.106	1.615	.903–2.891

Reference group: Working 36 months after the end of the rehabilitation program (binary logistic regression model)

N = 505 of 519 patients (due to listwise deletion); tolerance values between .605 und .991; Nagelkerke’s pseudo R^2^: 0.545

^a^Tumor stage was included in the multivariable analysis because it nearly reached statistical significance in bivariable analysis (*p* = .050)

Abbreviations: ß, unstandardized regression coefficient; SE, standard error; OR, odds ratio for independent variables; CI, 95% confidence interval; p, probability of type I error; t1, beginning of the rehabilitation program: t2, end of the rehabilitation program; HADS, Hospital Anxiety and Depression Scale; EORTC QLQ-C30, European Organization for Research and Treatment of Cancer Quality of Life - core questionnaire; AVEM, Occupational Stress and Coping Inventory; SIBAR, Screening Instrument Work and Occupation

Significant values are marked in bold

## Discussion

We analyzed long-term RTW and prospective predictors for not working in 519 LPCa survivors 3 years after post-acute rehabilitation following RP. The majority (88%) had started the rehabilitation program within 3 months after diagnosis of LPCa.

Our findings show that a substantial proportion of LPCa survivors (73%) worked at 36 months follow-up. This finding corresponds with a recent review on work retention of cancer survivors from mixed cancer sites, which found proportions of 72% working at 2–2.9 years post-diagnosis [12]. In our study, the most common reason for not working was early retirement (22%), followed by unemployment (4%), and having been granted a disability pension (1%). In comparison, a German population-based study investigating RTW outcomes of cancer survivors (mixed cancer sites) reported disability pension (17%), early retirement (6%), and unemployment (4%) as most frequent reasons for not working after an average of 8.3 years post-diagnosis [3].

Regarding patterns of occupational activity (12 and 36 months follow-up), continuous employment was observed in nearly three quarter of LPCa survivors (71%), while subgroups did not work at both time points (9%) or experienced unfavorable change of work status (18%). In a Dutch study, survivors from mixed tumor types (up to four years after diagnosis) were classified as “continuously working” in 60%, “continuously not working” in 20%, and “negative change in work status” in 15% [15]. Due to specific vs. mixed cancer sites and unknown proportion of the Dutch survivors who had undergone rehabilitation, comparability of data is limited but points to the fact that long-term work continuation is a realistic goal among cancer survivors including men treated by RP for LPCa.

Concepts of rehabilitation interventions addressing RTW after cancer vary considerably: Reviews point to different time points and localizations [6] and confirm that only diverse, multidisciplinary rehabilitation was effective compared with care as usual [39]. The sustaining high RTW rates in our study may reflect the beneficence of multidisciplinary approaches and early onset of rehabilitation in the RTW process of LPCa survivors treated by RP. However, we cannot attribute the positive RTW outcome to rehabilitation alone in the absence of a control group. A German population-based study including both rehabilitants and non-rehabilitants found no significant association between participation in oncological rehabilitation and RTW in the subgroup of prostate cancer survivors, but the observational design of this study also limits the information value regarding the potential effects of rehabilitation on RTW [3].

Regarding the predictor profile, our study showed older age and lower socio-economic status to increase the likelihood of not working among LPCa survivors. These findings correspond with previously reported prognostic factors for work retention in cancer survivors, both across cancer sites [3, 12] and specific to prostate cancer [27, 47]. Although not being modifiable, assessment of these factors is highly relevant since it allows for early identification of LPCa patients at risk and enables healthcare providers for timely planning of tailored rehabilitation measures. Special attention must be paid to the role of social inequality in the group of cancer survivors under study, and need for more research on work-related issues among cancer survivors with a lower socio-economic status has already been acknowledged [2, 10].

In contrast, fatigue and work behavior patterns, which also significantly predicted not working in our study, are modifiable factors. Fatigue has impact on cancer survivors’ physical and psychosocial functioning [2] and poses a relevant burden at work. Empirical evidence shows that fatigue is associated with unemployment [28], ability to retain paid employment [36], diminished work ability [37, 46] and work-related cognitive functioning [14, 24], and cancer survivors narrate symptoms to be difficult to manage at work. A recent review on work after prostate cancer also identified treatment-related side effects, including fatigue, as a barrier for reduced work status, longer sickness absence, and early retirement in this patient group [27]. Due to age-related cognitive and physical abilities, fatigue may even aggravate work-related problems in older LPCa survivors. However, it is notable that the strength of association between fatigue levels at the end of the rehabilitation program and not working 3 years post-rehabilitation was rather small. Interestingly, other common side effects of treatment did not impact RTW at 36 month follow-up. Neither patient-reported urinary incontinence, bowel symptoms, hormonal-therapy induced symptoms, and bother due to incontinence aid (assessed via EORTC-PR25 questionnaire) nor physician-reported extent of urinary incontinence at the end of rehabilitation were associated with the outcome. Prior studies indicated relationships particularly between urinary incontinence and work status [47]; however, follow-ups covered a shorter time period which limits comparability.

Regarding work behavior patterns, 43% of LPCa survivors not working 3 years post-rehabilitation had reported unambitious and 20% resigned work behavior during the rehabilitation program. While both behavior patterns include unambitious attitudes towards work, the first is further characterized by low engagement but positive emotionality, and the latter by lack of emotional distance at work, diminished stress resistance, and negative emotionality [31, 33]. Concurrently, previous research suggested that long-term cancer survivors who applied active coping mechanisms felt in a better position to handle work-related problems [16]. Since behavioral determinants might be of relevant impact on RTW retention, rehabilitation interventions should specifically address this topic to strengthen LPCa survivors’ resources. However, as it has already been concluded, impact and mechanisms of behavioral determinants need to be understood in greater detail [16].

Fatigue and psychosocial issues of cancer survivors have been confirmed to be amenable to cancer rehabilitation [35]. Treatment of fatigue symptoms and psychosocial interventions to support coping with the disease, treatment and its sequelae, including work-related issues, are central responsibilities of multidisciplinary cancer rehabilitation in Germany. Work-related medical rehabilitation programs have been strongly promoted by the German pension insurance in recent years; however, studies of interventions to improve occupational outcomes in cancer survivors have shown mixed results [7, 17].

Our study has several strengths including its longitudinal design, the well-defined sample of LPCa survivors and consistently high response rates. However, some methodological limitations need to be noticed. With regard to generalizability and interpretation of results, our findings cannot be interpreted for prostate cancer survivors who do not use rehabilitation services. As non-rehabilitants were not included as comparison group, it is not possible to directly proof the effects of rehabilitation on long-term RTW. Further, we only included LPCa survivors who had undergone RP, thus representing a cancer survivor group with relatively good prognosis. Together with the selection criteria of paid work prior RP, this might have resulted in an overestimation of the long-term RTW rate. Lacking information about rehabilitation participants who eventually died during the two follow-ups and about other reasons for nonresponse also restricts the interpretation of our findings. Furthermore, any conclusions regarding the role of fatigue in this study must be treated with caution because fatigue was measured as one symptom of the EORTC Quality of Life questionnaires only. As other studies with LPCa survivors treated by RP show that clinically relevant fatigue levels are less in this population than in other treatment groups [4, 38], it would be highly recommendable that future studies measure and explore the role of fatigue in this specific patient population more comprehensively, to resolve any inconsistencies in this regard.

## Conclusion

Sustained RTW is a realistic goal for many LPCa survivors after RP who had attended a post-acute cancer rehabilitation program. The results of this study contribute to the identification of at-risk LPCa survivors early in the RTW process. They point to the need for tailored rehabilitation to avoid marginalization of those with low social status due to long-term labor market withdrawal. Further, supportive interventions related to the management of fatigue symptoms and work-related coping styles during the course of rehabilitation and aftercare could improve RTW in the population under study, as these emerged as significant but modifiable factors impeding work retention. Adequate screening is needed to target the subgroup of LPCa survivors in need of intensified or specifically tailored care. This could include the application of existing, validated instruments for assessment of work behavior patterns, and coping styles.

## Data Availability

The authors have full control over the primary data. The data analyzed in this study are housed at the Department of Medical Psychology, University Medical Center Hamburg-Eppendorf, Martinistr. 52, 20,246 Hamburg, Germany. As per the research ethics committee approval, this dataset is subject to ethical restrictions and local data protection regulations that do not allow publication of raw data. All relevant data for the conclusions are presented in the manuscript.
